# Update on the Cardiac Safety of Moxifloxacin

**DOI:** 10.2174/157488612802715735

**Published:** 2012-04

**Authors:** Wilhelm Haverkamp, Frank Kruesmann, Anna Fritsch, David van Veenhuyzen, Pierre Arvis

**Affiliations:** 1 Department of Cardiology, Campus Virchow Clinic, Charité University Medicine Berlin, Berlin, Germany; 2 Bayer Pharma AG, Wuppertal, Germany; 3 Bayer HealthCare, Montville, NJ, USA; 4 Bayer HealthCare, Loos, France

**Keywords:** Cardiac safety, comparator antimicrobials, moxifloxacin, oral therapy, Phase II–IV clinical trials, QTc prolongation, sequential intravenous/oral therapy.

## Abstract

Cardiac safety was compared in patients receiving moxifloxacin and other antimicrobials in a large patient population from Phase II–IV randomized active-controlled clinical trials. Moxifloxacin 400 mg once-daily monotherapy was administered orally (PO) or sequentially (intravenous/oral, IV/PO). Across 64 trials, 21,298 patients received PO therapy (10,613 moxifloxacin, 10,685 comparators) while 6846 received sequential IV/PO therapy (3431 moxifloxacin, 3415 comparators). Treatment-emergent cardiac adverse event (AE) rates were similar for moxifloxacin and comparators in PO (6.6% *vs* 5.8%) and IV/PO (11.0% *vs* 12.0%) trials. Treatment-emergent cardiac adverse drug reactions were rare in PO (moxifloxacin 3.2% *vs* comparators 2.4%) and IV/PO (moxifloxacin 1.4% vs comparators 1.5%) patients. There were five (<0.02%) treatment-emergent drug-related deaths due to cardiac events out of 28,144 patients; one PO patient died treated with comparators, one patient died treated with IV/PO moxifloxacin, and three patients died after treatment with IV/PO comparators. Only one case of treatment-related non-fatal *torsade de pointes* occurred in the comparator arm. Incidence rates of cardiac AEs remained low in populations at elevated risk of cardiac events predisposed to QTc prolongation (i.e. community-acquired pneumonia patients admitted to the intensive care unit and/or mechanical ventilation, patients with documented prolongation of baseline QTc interval, women, and patients ≥ 65 years old). There was no evidence of unexpected cardiac events. After moxifloxacin treatment, an expected small prolongation in QTcB and QTcF was found. This analysis of numerous clinical trials shows the favorable cardiac safety profile of moxifloxacin, when used appropriately and according to its label, versus other antibiotics.

## INTRODUCTION

Many non-anti-arrhythmic drugs, such as antipsychotics (e.g. pimozide, sertindole, ziprasidone, quetiapine, haloperidol, thioridazine) [1-4], antihistamines (e.g. astemizole, terfenadine) [3], and antimicrobials/antimalarials (e.g. erythromycin, ketoconazole, chloroquine, halofantrine) [3-6] are known to prolong the cardiac repolarization phase, i.e. the QT interval of the surface electrocardiogram. Prolonged repolarization has the potential to increase the risk of cardiac events such as ventricular arrhythmias [1], including the potentially life-threatening ventricular tachycardia known as *torsade de pointes *(TdP) [3, 7], and cardiac arrest.

QT prolongation has been observed with several classes of antimicrobials [7], including macrolides (e.g. clarithromycin, erythromycin) [7], ketolides (telithromycin) [7, 8], and fluoroquinolones (e.g. ciprofloxacin, levofloxacin, moxifloxacin, ofloxacin, sparfloxacin) [7, 9-13]. The potential to cause TdP appears to be higher for macrolides, when given intravenously, than for fluoroquinolones [7, 14]. Within each class, the agents seem to differ in their potential to cause such cardiac events [15].

It is well established that moxifloxacin produces a predictable small prolongation of the corrected QT (QTc) interval [11] by reversible and dose-dependent but weak blockage of the rapidly activating delayed rectifier potassium channel, I_Kr_ [16] or human cloned counterpart, the hERG potassium channels [17]. The predictability of moxifloxacin-induced QTc changes has led to its use as a positive control in thorough Phase I QT/QTc studies [18, 19]. However, despite the observed QTc prolongation with moxifloxacin, there is no evidence that its use is associated with an increase in the overall number of cardiac events [11, 20-24]. Even in a high-risk population of elderly patients with community-acquired pneumonia (CAP), sequential intravenous/oral (IV/PO) treatment with moxifloxacin has a good cardiac rhythm safety profile, comparable to that of levofloxacin [25].

The objective of this analysis was to investigate the cardiac safety of moxifloxacin and to compare it with a range of other antimicrobials in a large patient population. The data presented here cover cardiac safety findings from 64 of the 65 Bayer-sponsored Phase II to IV randomized, active-controlled clinical trials with moxifloxacin 400 mg, including indications requiring oral (PO) administration and those where sequential IV/PO therapy could be used. One Phase II trial included only patients with cirrhotic liver disease, and was excluded from the analysis due to enrolment of very different patients compared with other studies (most patients had a Child-Pugh C cirrhosis, which is a contraindication for the use of moxifloxacin). Several of these clinical trials included electrocardiographic (ECG) recording, providing a large patient subpopulation with documented QTc intervals. A secondary objective, therefore, was to confirm the known QTc prolongation with moxifloxacin and to determine the variability of QTc intervals in the patients included in randomized active-controlled clinical trials with moxifloxacin.

## PATIENTS AND METHODS

### Studies Included in the Analysis

A pooled analysis was carried out using data from Phase II to IV clinical trials comparing moxifloxacin with other antimicrobials.

In all studies, 400 mg of moxifloxacin was administered once daily as monotherapy either PO or sequentially (IV/PO). The PO indications for moxifloxacin were acute bacterial sinusitis (ABS, n = 10 trials), acute exacerbations of chronic bronchitis (AECB, n = 17), CAP (n = 12), uncomplicated skin and skin structure infections (uSSSI, n = 4), uncomplicated pelvic inflammatory disease (uPID, n = 3), uncomplicated urinary tract infections (uUTI, n = 3), complicated urinary tract infections (cUTI, n = 1), and streptococcal pharyngitis (n = 1). The IV/PO trials for moxifloxacin were carried out in patients hospitalized with CAP (n = 7), complicated skin and skin structure infections (cSSSI, n = 3), complicated intra-abdominal infections (cIAI, n = 2), hospital-acquired pneumonia (HAP, n = 2), and aspiration pneumonia or lung abscess (n = 1). A few patients with related indications could be enrolled in two studies, for instance AECB and ABS, and AECB and CAP. In each trial, appropriate PO or IV antimicrobials licensed for the relevant indication were used as the comparator agents; the route of treatment administration may have differed between moxifloxacin and comparator within single trials, as seen in Table **1**. Control data are for pooled comparator agents. All control comparators were used at the recommended dose and for the recommended duration – based on the relevant labels at the time and in the region (when and where) the clinical study was conducted – in the applicable indication and adverse events (AEs) were monitored and recorded according to the same standards in all studies, thereby justifying pooling of data in the analysis.

### Cardiac Safety Populations and Assessments

All analyses were performed for the population of patients valid for safety. This included all randomized patients who had received at least one dose of study drug and had one safety assessment. Given the clinical heterogeneity of the PO and IV/PO populations, the two data sets were analyzed separately. All data available in the Bayer HealthCare clinical trial database up to a data lock point of 31 May 2009 were included.

Standard safety outcomes recorded in the individual clinical trials were coded using the Medical Dictionary for Regulatory Activities (MedDRA, version 12.0). Cardiac events were defined according to MedDRA preferred terms (PTs) identified using the following Standardized MedDRA Queries: (i) sets of PTs with a primary or secondary path in the System Organ Class ‘Cardiac disorders’; and (ii) sets of PTs that were considered potential surrogates for TdP/QTc prolongation. The MedDRA events (PTs) included as potential surrogates for TdP/QTc prolongation were: cardiac arrest, cardiorespiratory arrest, cardioversion, sudden cardiac death, cardiac death, cardiac flutter, cardiac massage, cardiac fibrillation, death, sudden death, brain death, maternal death affecting fetus, syncope, loss of consciousness, resuscitation, anoxic seizure, TdP, ventricular arrhythmia, ventricular asystole, ventricular fibrillation, ventricular flutter, and ventricular tachycardia.

The following safety variables were considered for analysis: treatment-emergent cardiac AEs, treatment-emergent drug-related cardiac AEs, treatment-emergent serious cardiac AEs, treatment-emergent serious drug-related cardiac AEs, treatment-emergent cardiac AEs with fatal outcome, and treatment-emergent drug-related cardiac AEs with fatal outcome. AEs considered as potential surrogates for TdP/QTc prolongation were also analyzed.

Treatment-emergent AEs were defined as any AE occurring after the first administration of study drug until the end of follow-up.

Cardiac safety variables were analyzed for the overall safety population and two potentially high-risk subgroups with characteristics known to be independently associated with QTc prolongation. These subgroups were women [26-28] and patients of at least 65 years of age [29].

Treatment-emergent events considered to be potential surrogates for TdP/QTc prolongation were assessed in the overall safety population and further subpopulations at high risk of cardiac arrhythmias and other cardiac AEs: (i) patients on initial IV treatment with CAP requiring admission to the intensive care unit (ICU) and/or mechanical ventilation, (ii) patients with a documented prolonged QTcB interval at baseline (defined as > 450 ms in men or > 470 ms in women), (iii) women, and (iv) patients ≥ 65 years old.

ECG recordings were carried out in several of the randomized active-controlled trials in approved indications for moxifloxacin that were included in the pooled analysis, including two IV/PO cSSSI studies, five IV/PO CAP studies, and 11 PO ABS, AECB, CAP, cUTI, uPID, and uSSSI studies. The studies included had centralized ECG readings and a standardized methodology was used. QTc data presented here are for patients with valid ECG records (Supplementary material) at baseline, Day 1 and Day 3 (Group A), at baseline and on Day 1 (Group B), or baseline and Day 3 (Group C) of treatment while on IV therapy for CAP or cSSSI. They are presented for patients with paired valid ECG records at baseline and post-baseline in the PO clinical studies. QTc data are available for the overall valid population and for subgroups at higher risk of cardiac events (i.e. women and patients ≥ 65 years old). ECG recordings were planned to occur at expected peak plasma concentrations of the antimicrobial (*C*_max _i.e. PO: approx. 3 h after administration, IV: within 30 min after the end of a 60 minute-infusion). QTcB (QT interval corrected for heart rate and calculated according to Bazett’s formula) and QTcF (QT interval corrected for heart rate and calculated according to Fridericia’s formula) are presented. Of note, both the European Medicines Agency (EMA) and the US Food and Drug Administration (FDA) require submission of QTc values corrected by using both Bazett and Fridericia formulae.

## RESULTS

### Demographics

A total of 29,548 patients were enrolled in 64 trials (49 PO, 15 IV/PO) of moxifloxacin versus comparator antimicrobials. Of these, 28,144 patients were included in the pooled safety population; 21,298 had received PO antimicrobials (10,613 moxifloxacin, 10,685 comparators) and 6846 had received sequential IV/PO antimicrobials (3431 moxifloxacin, 3415 comparators).

Within the PO group or the IV/PO group there were no relevant differences in patient characteristics between moxifloxacin and comparator groups (Table **2**). However, there were differences in characteristics between patients given PO or IV/PO antimicrobials. In the PO group, most patients had received outpatient antimicrobial treatment for the respiratory indications of ABS, AECB, and CAP, while in the IV/PO group, most patients were hospitalized with either CAP or cSSSI. Patients in the PO group were about 8 years younger than those in the IV/PO group, with 22% in the PO group and 40% in the IV/PO group being ≥ 65 years of age. There were also more women in the PO group than the IV/PO group (54% *vs* 39%, respectively). Finally, in both treatment arms patients in the IV/PO group compared with those in the PO group were more likely to be taking comedications known to cause QT prolongation (IV/PO: MXF: 10.0%, COMP: 8.7% *vs* PO: MXF: 4.1%, COMP: 4.0%, respectively) and to have cardiac disease (IV/PO: MXF: 34.0%, COMP: 33.3% *vs* PO: MXF: 13.9%, COMP: 13.1%, respectively). There were some differences in heart rates at baseline (Tables **2** and **7**). IV/PO CAP patients presented with the highest mean baseline heart rate values (moxifloxacin 90.5 beats per minutes [bpm], comparators 90.9 bpm), followed by those with IV/PO cSSSI patients (moxifloxacin 82.2 bpm, comparators 80.2 bpm). Patients receiving PO therapy had the lowest heart rates (moxifloxacin 77.2 bpm, comparators 77.0 bpm).

### Overall Incidence of Cardiac AEs

#### Treatment-Emergent AEs and Treatment-Emergent Serious AEs

There was no evidence of any excess of cardiac AEs with moxifloxacin versus comparators in the PO or IV/PO populations (Table **3**). In the overall safety population, the number of patients with treatment-emergent cardiac AEs was low for both treatment arms irrespective of route of treatment administration. Of patients receiving PO therapy, 698/10,613 (6.6%) in the moxifloxacin arm and 619/10,685 (5.8%) in the comparator arm experienced a treatment-emergent cardiac AE. In those receiving IV/PO therapy, equivalent numbers were 377/3431 (11.0%) for moxifloxacin and 410/3415 (12.0%) for comparators. Treatment-emergent serious cardiac AEs also occurred in low numbers both in the PO (moxifloxacin: 75/10,613 [0.7%]; comparators 72/10,685 [0.7%]) and IV/PO groups (moxifloxacin 117/3431 [3.4%], comparators 118/3415 [3.5%]).

In women and patients over 65 years old (i.e. at higher risk of cardiac arrhythmias and other cardiac AEs), rates of treatment-emergent cardiac AEs were higher for IV/PO studies (13.2–18.3%) than for PO studies (6.1–8.6%), as observed in the overall population. Rates of serious cardiac AEs were also higher for IV/PO studies (3.7–6.2%) versus PO studies (0.4–1.7%). A similar percentage of at-risk patients in each treatment arm experienced a treatment-emergent AE or serious cardiac treatment-emergent AE (Table **3**).

#### Treatment-Emergent Drug-Related AEs and Treatment-Emergent Drug-Related Serious AEs

Treatment-emergent drug-related AEs are summarized in Table **3**. In the PO group, 342/10,613 (3.2%) of moxifloxacin- and 256/10,685 (2.4%) of comparator-treated patients experienced a treatment-emergent drug-related cardiac AE. Equivalent numbers for the IV/PO group were 49/3431 (1.4%) for moxifloxacin and 50/3415 (1.5%) for comparator treatments. There were no clinically relevant differences in any type of treatment-emergent cardiac AEs between moxifloxacin and comparators in either the PO or IV/PO group. Treatment-emergent serious drug-related cardiac AEs were reported for 7 PO patients (< 0.1%) in the moxifloxacin group, and 6 (< 0.1%) in the comparator group, and for 6 (0.2%) versus 11 (0.3%) of IV/PO patients in the moxifloxacin and comparator arms, respectively.

In women, there were no major clinically relevant differences between moxifloxacin and comparators in either the PO or IV/PO group with respect to the incidence of treatment-emergent drug-related cardiac AEs. In patients ≥ 65 years old, the only difference seen was in drug-related treatment-emergent AEs in PO studies. These were more frequent in the moxifloxacin than the comparator group (76/2451, 3.1% *vs* 36/2403, 1.5%, respectively), with the difference driven by a higher occurrence of dizziness (moxifloxacin 61/2451, 2.5% *vs* comparators 21/2403, 0.9%).

#### Treatment-Emergent AEs and Drug-Related AEs with Fatal Outcome

Treatment-emergent cardiac AEs with a fatal outcome were rare in the PO and IV/PO groups. In PO patients, there were 12 (0.1%) treatment-emergent cardiac AEs with a fatal outcome with moxifloxacin and 21 (0.2%) with comparators. Of these, none with moxifloxacin and only one (< 0.1%) with a comparator were considered drug-related. This patient had septicemia and died from cardiorespiratory failure. In the IV/PO group, there were 43 (1.3%) versus 44 (1.3%) treatment-emergent cardiac AEs with a fatal outcome for moxifloxacin and comparators, respectively. Of these, one with moxifloxacin and three with comparators were considered drug-related. The moxifloxacin-treated patient died after having ventricular tachycardia in the context of a myocardial infarction leading to hypotension and acute respiratory failure. Causes of death in the comparator arm were cardiac arrest secondary to bilateral lung infiltrates, acute myocardial infarction and acute cardiorespiratory failure, and ventricular tachycardia.

The incidences of treatment-emergent cardiac AEs with fatal outcome in women were similar for moxifloxacin and comparators in both the PO (3 [0.1%] moxifloxacin, 4 [0.1%] comparators) and IV/PO (19 [1.4%] moxifloxacin, 15 [1.1%] comparators) groups. Only one of these events in the IV/PO group (comparators) was considered drug-related. Incidences of treatment-emergent cardiac AEs with fatal outcome were also similar in patients ≥ 65 years in both the PO (9 [0.4%] moxifloxacin, 12 [0.5%] comparators) and IV/PO (32 [2.3%] moxifloxacin, 37 [2.8%] comparators) groups. Three events in the IV/PO group, all receiving comparator treatment, were considered drug-related.

### Incidence of Cardiac AEs Considered Potential Surrogates for TdP/QTc Prolongation

The overall incidence of treatment-emergent cardiac AEs considered potential surrogates for TdP/QTc prolongation was lower in the PO group than the IV/PO group, probably reflecting differences in severity of illness and underlying conditions (Table **4**). There was no evidence of any excess of cardiac AEs with moxifloxacin versus comparators in either the PO or IV/PO groups.

In addition, the incidence of specific cardiac events was similar in the moxifloxacin and comparator groups. Across the PO and IV/PO groups, 14 moxifloxacin- and 14 comparator-treated patients developed a ventricular arrhythmia, ventricular fibrillation, or ventricular tachycardia. Ten of these cases were drug-related (6 moxifloxacin, 4 comparators). Cardiac arrest or cardiorespiratory arrest was reported in 16 moxifloxacin- (PO 3; IV/PO 13) and 18 comparator-treated (PO 2; IV/PO 16) patients, the majority of them being treated for CAP, HAP, or aspiration pneumonia. Of the 16 moxifloxacin-treated patients, six had existing cardiac comorbidity, two had an underlying respiratory disease, and eight had comorbidities including hypertension, diabetes, renal impairment, and/or alcohol abuse. In the comparator arm, 13 patients had an existing cardiac comorbidity, three had underlying respiratory disease, and two had both cardiac comorbidities and underlying respiratory disease.

There was only one case of TdP reported in the clinical studies; this occurred in a patient treated with an IV/PO comparator agent. This elderly patient, who was enrolled in a CAP study, was administered levofloxacin 500 mg once daily.

Looking specifically at treatment-emergent events considered potential surrogates for TdP/QTc prolongation and the incidence of loss of consciousness and syncope, numbers were low and similar for moxifloxacin and comparator agents given either PO or IV/PO (Table **4**). Cardiac AEs that were potential surrogates for TdP/QTc prolongation and had a fatal outcome were reported in 8/10,613 moxifloxacin patients and 6/10,685 comparator-treated patients in the PO group and 13/3431 moxifloxacin- *vs* 17/3415 comparator-treated patients in the IV/PO group.

Of these events, one in the PO comparator group was considered drug-related (death following respiratory arrest as the consequence of septicemia), while in the IV/PO group, one in the moxifloxacin arm (ventricular tachycardia as a complication of acute myocardial infarction and respiratory failure) and two (cardiorespiratory arrest in two patients) in the comparator groups were considered drug-related.

### Incidence of Cardiac AEs Considered Potential Surrogates for TdP/QTc Prolongation in High-Risk Subsets of Patients

Four high-risk subsets of patients were included in this analysis: patients on initial IV treatment with CAP requiring admittance to the ICU and/or mechanical ventilation, patients with a prolonged baseline QTcB interval (> 450 ms in men, > 470 ms in women), women, and patients ≥ 65 years old.

Of the 6662 patients with CAP in the safety population, 316 had CAP requiring ICU admission and/or mechanical ventilation and initial IV treatment (149 moxifloxacin, 167 comparators). Cardiac AE profiles for these patients were similar in the moxifloxacin and comparator groups (Table **5**) and no deaths were observed in either group during follow-up. Treatment-emergent cardiac AEs were reported in 55 (36.9%) moxifloxacin- and 59 (35.3%) comparator-treated patients. Looking specifically at treatment-emergent cardiac AEs considered potential surrogates for TdP/QTc prolongation, seven (4.7%) were reported for moxifloxacin-treated patients and eight (4.8%) for comparator-treated patients (Table **5**).

Three groups of patients with a prolonged QTcB interval at baseline were assessed: PO patients, IV/PO CAP patients and IV/PO cSSSI patients. Among the 145 PO patients with data available (71 in the moxifloxacin group and 74 in the comparator group), there were two treatment-emergent cardiac AEs considered potential surrogates for TdP/QTc prolongation in each group: cardiac flutter and syncope in the moxifloxacin group and cardiac flutter and loss of consciousness in the comparator group. No deaths were observed. No events were seen in the IV/PO cSSSI population, while in the IV/PO CAP population (n = 221 with available data) there was one event in the moxifloxacin group (sudden death) and two in the comparator group (cardiac arrest and ventricular tachycardia).

Of the 14,262 women enrolled in the study, 21 (10 moxifloxacin, 11 comparators) in PO studies and 22 (11 moxifloxacin, 11 comparators) in IV/PO studies experienced treatment-emergent cardiac AEs considered potential surrogates for TdP/QTc prolongation, with similar cardiac AE profiles being observed between arms (Table **6**). In the IV/PO group there was one death (moxifloxacin) and two sudden deaths (comparator group) reported in women patients.

In the 7561 patients ≥ 65 years old, treatment-emergent cardiac AEs considered potential surrogates for TdP/QTc prolongation were experienced by 16 (9 moxifloxacin, 7 comparators) in PO studies and 46 (24 moxifloxacin, 22 comparators) in IV/PO studies, with no apparent differences between groups. Two deaths (moxifloxacin IV/PO group), one sudden cardiac death (moxifloxacin IV/PO group) and six sudden deaths (PO: 1 moxifloxacin, 1 comparators; IV/PO: 2 moxifloxacin, 2 comparators) occurred in the subgroup of patients ≥ 65 years old.

### Absolute QTcB and QTcF Values at Baseline and Changes after PO and IV/PO Treatment with Antimicrobials

**Fig. (2):**
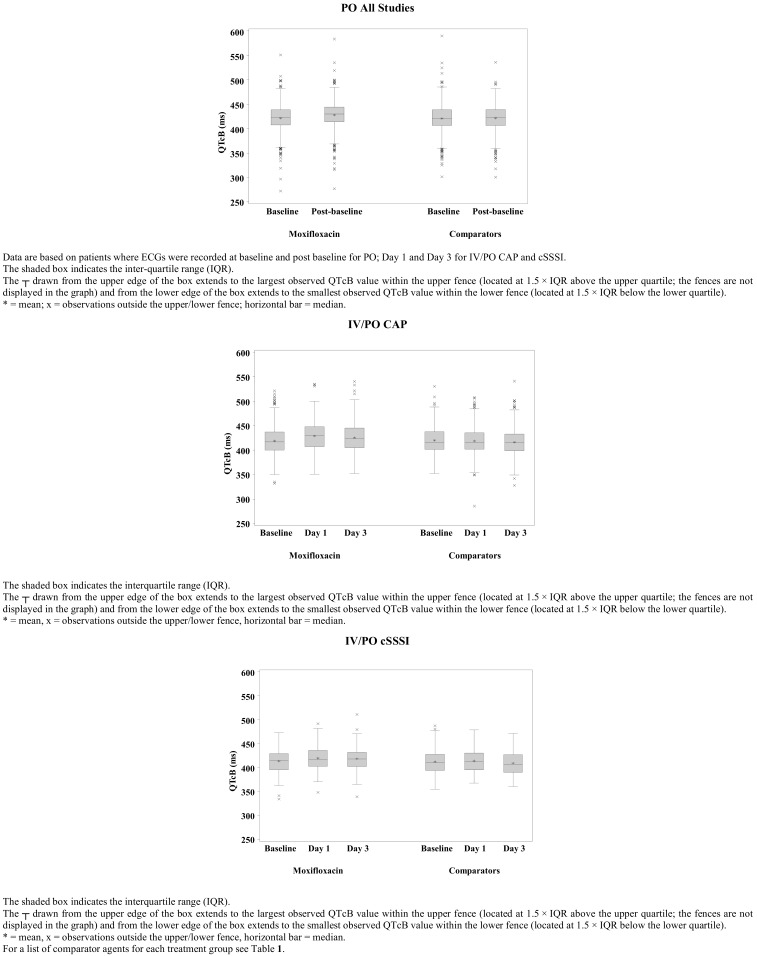
Boxplots for QTcB Intervals in Randomized Active-Controlled Phase II to IV Studies where ECG was Recorded at Baseline and Post baseline (PO) and Baseline, Day 1 and Day 3 (IV/PO) of Treatment with Moxifloxacin or Comparators.

Disposition of patients with ECG recordings at baseline and post-baseline is presented in Fig. (**1**) for the three populations outlined above (PO, IV/PO CAP, and IV/PO cSSSI). There was wide overlap in the interquartile range for all populations and a wide range in individual QTcB intervals both at baseline and post-baseline, with minimum QTcB values of 273–352 ms and maximum values of 492–590 ms for PO and IV/PO populations (Fig. **2**).

Baseline QTcB data for all three populations (PO, IV/PO CAP, and IV/PO cSSSI) were similar with mean QTcB intervals for both treatment arms in the range of 414–421 ms with a standard deviation (SD) of about 27 ms (Table **7**). In addition to QTcB values, baseline QTcF values were also found to be similar among the two treatment groups for each population (in the range of 391–405 ms and a standard deviation of about 27 ms). QTcF values at baseline were lower than corresponding QTcB values in all groups (the overcorrection on heart rates associated with the Fridericia’s formula being probably less pronounced than that of the Bazett’s formula) and slightly higher in the PO studies than in the IV/PO studies in both treatment arms; a difference possibly occurring due to higher heart rates in the hospitalized patients treated with sequential IV/PO therapy.

As expected, moxifloxacin produced a small mean QTcB prolongation (Table **7**). In PO studies post-baseline prolongation in the order of 6.4 ms was seen, while in IV/PO studies the average prolongation ranged from 6.1 to 10.1 ms on Day 1 post-dose and from 4.4 to 6.6 ms on Day 3 post-dose. In contrast, a mean 0.6 ms change was seen post-dose in the PO comparator treatment group, while in IV/PO studies prolongation ranged from -1.3 to 1.8 ms on Day 1 post-dose and from -3.9 to -3.2 ms on Day 3 post-dose.

Calculation of QTcF confirms the QTcB data in the PO group, where the QTc change from baseline to post-baseline was similar when calculated as QTcB or QTcF with both moxifloxacin (6.4 ± 25.5 ms *vs* 7.5 ± 24.8 ms, respectively) and comparators (0.6 ± 23.1 ms *vs* 2.0 ± 21.8 ms) (Table **7**). Larger differences were seen between QTcF and QTcB-calculated values in hospitalized patients with tachycardia in the IV/PO CAP group. Here, in group C the change from baseline to Day 3 with moxifloxacin treatment was 13.3 ± 25.6 ms when calculated using QTcF and 5.7 ± 26.3 ms when calculated using QTcB. Corresponding values for comparators were 4.3 ± 26.8 ms and -3.5 ± 27.6 ms, respectively. In the IV/PO cSSSI group, the change from baseline to Day 3 in group C with moxifloxacin treatment was 9.9 ± 20.1 ms when calculated using QTcF and 4.4 ± 21.5 ms when calculated using QTcB. Corresponding values for comparators were 1.8 ± 17.3 ms and -3.6 ± 20.2 ms, respectively. A similar trend was observed in group A patients on Day 3 in IV/PO CAP, and IV/PO cSSSI studies, also in both treatment groups. Concerning those patients in group B and group A on Day 1 in the sequential IV/PO studies, no differences were seen between QTcF and QTcB-calculated values in either treatment arm.

### QTcB Changes in Subgroups at Higher Risk of Cardiac AEs

In women and patients ≥65 years old, there was some variation in the extent of QTcB changes. Changes in the moxifloxacin group ranged from 6.2 to 12.7 ms in women and from 1.8 to 12.4 ms in patients ≥65 years (Table **8**). Conversely, in the comparator group, changes in women ranged from -8.7 to 1.5 ms and from -4.0 to 2.8 ms in patients ≥65 years (Table **8**). In moxifloxacin-treated patients of group A, although QT prolongation after the first IV dose (on Day 1) was slightly higher in the IV/PO CAP population than in the IV/PO cSSSI population for both women (12.7 *vs* 7.4 ms, respectively, in Group A) and patients ≥65 years old (12.4 *vs* 8.2 ms), QTcB changes by Day 3 in the IV/PO CAP group were similar to the post-baseline levels seen in the PO population (7.8 ms in women and 7.6 ms in patients ≥65 years old), and only 1–2 ms higher than that observed in the overall study population (6.6 ms). Changes from baseline to Day 1 in group B patients and from baseline to Day 3 in group C patients were similar to those seen in group A on Day 1 and Day 3, respectively.

## DISCUSSION

The major finding of the analysis was that moxifloxacin treatment resulted in an expected small prolongation of the QTc interval which did not translate into an increased risk of TdP or other ventricular arrhythmias. This data is in agreement with a recent assessment carried out by the EMA PhVWP confirming that moxifloxacin has a potential to prolong the QTc interval in patients [30]. The low potential of prolongation of the QTc interval has been considered greater for moxifloxacin than for other fluoroquinolones (i.e. ciprofloxacin, levofloxacin).

A mean prolongation at steady state of the ECG QTc interval by 4–7 ms is a well-known effect of moxifloxacin [11]. As with all drugs that affect the QTc interval [31, 32], there is the possibility that in rare cases a delay in cardiac repolarization could translate into serious cardiac events. For this reason, moxifloxacin is contraindicated or should be used with caution (depending on the countries) in patients with a range of pro-arrhythmic conditions [33, 34].

Individual clinical studies with moxifloxacin [11, 20-24] and an analysis of a large Phase II to IV data set (approximately 6000 patients) [35] have not found increased cardiac morbidity or mortality with moxifloxacin. The current analysis, which used extensive Phase II to IV randomized active-controlled clinical trials data for moxifloxacin, confirms the cardiac safety of moxifloxacin (PO and IV/PO) across a range of indications and in comparison with commonly used antibiotics, as seen in previous studies. In this large population of patients, the incidences of treatment-emergent cardiac AEs were similar for moxifloxacin and comparators; the larger number of cardiac AEs seen in IV/PO versus PO patients in both groups is likely to be explained by an increased frequency of cardiac comorbidities and the severity of patients. There was a low incidence of drug-related treatment-emergent cardiac AEs with both moxifloxacin and comparators and only five drug-related treatment-emergent deaths due to cardiac AEs in the entire population, one treated with a comparator in the PO group, one with IV/PO moxifloxacin, and three with IV/PO comparator agents. The incidence of drug-related cardiac AEs was higher in the PO population than in IV/PO population in both treatment groups. However, patients receiving IV/PO treatment are more likely to have cardiac-related co-morbidities and underlying conditions that may affect the reporting of cardiac AEs. There was no reported case of TdP in 14,044 moxifloxacin-treated patients.

Even in patient populations at elevated risk of cardiac AEs due to the potential for underlying prolongations in QTc interval (i.e. women and those of least 65 years old), there was no evidence of any additional risk for treatment-emergent cardiac AEs.

The patient subgroup with serious CAP was defined by admission to the ICU and/or requirement for mechanical ventilation. Such very ill patients who require initial parenteral antimicrobial treatment would be expected to have more cardiac risk factors than the wider populations of patients treated with antimicrobials for indications such as ABS, AECB, mild-to-moderate CAP, and cSSSI. The cardiac AE profiles in this high-risk population were similar for moxifloxacin and comparator groups and the incidence of treatment-emergent cardiac AEs considered potential surrogates for TdP/QTc prolongation with moxifloxacin was low.

In the subset of patients with ECG measurements, the mean baseline QTcB interval was similar (about 420 ms) for moxifloxacin- and comparator-treated patients across PO and IV/PO indications. At each measurement point (baseline, Day 1, Day 3 [IV/PO] or post-baseline [PO] of antimicrobial treatment), there was wide between-patient variation in QTc intervals, as demonstrated by the large and overlapping SDs and ranges. The change in QTc from baseline was as expected for moxifloxacin [11, 19, 25, 36-39], with QTcB values up to 10 ms on Day 1 following the initial intravenous treatment and falling to 4–7 ms by Day 3 when steady state could be expected. The latter increase is similar to the post-baseline levels observed in the PO population. The increase in QTcB with moxifloxacin was higher after IV/PO than PO administration. This is likely to be explained by the fact that patients receiving initially IV moxifloxacin were more severely ill and had a higher baseline heart rate than those receiving PO moxifloxacin.

Much of the cause for discrepancy in correction factors is because no single mathematical transformation can describe the rapidly changing nonlinear dynamics of the QT-RR interval relationship. The relatively large differences observed at Day 3 post-baseline between the findings derived from the two correction formulas (QTcB and QTcF) in the IV/PO CAP and cSSSI studies for both moxifloxacin and comparator-treated patients are most likely related to the differences in heart rate at the different time points in these populations. Both the Bazett’s and Fridericia’s formulas over-correct at high heart rates with the effect being more pronounced with the Bazett’s formula [4, 40]. In patients where the heart rate decreases between time points (i.e. during the course of therapy), the overcorrection decreases in numerical terms with the Bazett’s formula compared to the Fridericia’s formula, and the absolute increase in QTc appears to be larger using the Fridericia’s formula than when using the Bazett’s formula. In comparator-treated patients, changes in QTcB from baseline were generally small and positive at Day 1 post-baseline, and small and negative (a shortening of the QTc interval) at Day 3 post-baseline. Post-baseline changes in comparator-treated patients were always positive (1–3 ms) when using the Fridericia’s formula. In subgroups generally thought to be more susceptible to abnormal QTc prolongation (women and those ≥ 65 years old), the QTcB prolongation with moxifloxacin was generally small and consistent with that seen in the overall population.

In this analysis, where ECG data were available, only small increases in QTc were observed. This did not translate into clinical cardiac events. That prolongation occurring at relatively low levels may be due to the lack of effect of moxifloxacin on ion channels other than I_Kr_/hERG such as I_Kr_/KvLQT1/min K channel, which is unaffected by moxifloxacin even at high concentrations [41]. The 4–10 ms prolongation in QTcB (and 6–14 ms in QTcF) observed with moxifloxacin falls well below the likely threshold value (approximately 60 ms) for eliciting a cardiac AE that may be a surrogate clinical marker of TdP [35, 42]. Similarly, a review of data from animal models indicates an absence of TdP when moxifloxacin was administered at clinically relevant dosages and confirms the low rate of cardiac AEs seen in the current analysis [43]. The predictable pharmacokinetic properties of the drug [16] and its lack of interaction with cytochrome P450 [44] may also contribute to the lack of cardiac events seen.

The results of the current study support those from extensive testing in preclinical studies, which showed that the QTc prolongation observed with moxifloxacin is generally small and highly reproducible [37-39], and independent of the sex, age, or race of the subject [19]. The small QTc interval-prolonging effect of moxifloxacin is now acknowledged by the regulatory agencies, and is implicitly recognized by the EMA, the US FDA, and the International Conference on Harmonisation of Technical Requirements for Registration of Pharmaceuticals for Human Use as a positive control in thorough QTc studies used to assess the QTc effect of new compounds in Phase I trials [42, 45]. Although this analysis lacks data from placebo-controlled clinical trials; the reproducibility and the degree of QTc prolongation induced by moxifloxacin makes the drug useful as a positive control in investigating the pro-arrhythmic potential of new non-cardiac drugs. Fluoroquinolones have different potency of inhibiting the I_kr_/hERG potassium channel and as expected, prolongation of the QT interval consequently matches their IC_50_ value when they are administered at their recommended doses. The potential for QTc prolongation associated with moxifloxacin may be higher than that for the other fluoroquinolones currently on the market. However recent reviews of available preclinical and clinical studies concluded that the overall risk of TdP associated with these agents including moxifloxacin is small [30, 46].

Simply observing a prolongation of QTc interval does not necessarily predict the risk of ventricular arrhythmias [25] because, although the risk of developing arrhythmias is related to the length of the QTc interval, there is a high degree of QTc interval variability [47]. In addition, many independent variables (e.g. female sex, age ≥ 65 years, bradycardia, hypokalemia, hypocalcemia, hypomagnesemia, history of cardiac disease, and concomitant QTc-prolonging medication [48-51]) markedly affect the propensity to the development of cardiac events such as TdP [52]. It is important, therefore, to assess both the ECG changes and the impact of drug-induced QTc prolongation on clinically relevant safety end points. In general, drug-induced ventricular arrhythmias have been associated with excessive QTc interval prolongation (i.e. ≥ 500 ms) [14]. The clinical significance of non-cardiac medications that induce small increases in QTc interval (≤ 10 ms) remains controversial. More moxifloxacin-treated patients than comparator-treated patients had post-baseline QTcB ≥500 ms and/or ∆ QTcB ≥60 ms (for example, in IV/PO studies, 22 [2.3%] *vs* 11 [1.1%] patients had ∆ QTcB ≥60 ms in the population which had ECG recorded pre-therapy and on Day 3). This difference is not unexpected since moxifloxacin is known to prolong the QTc interval in some patients. Interestingly, there was no increased risk of cardiac adverse events among these patients.

Whether a correlation exists between plasma concentrations of moxifloxacin and prolongation of QTcB is still a matter of debate. Although no significant correlation was seen in a Phase III study enrolling patients with paired-valid ECGs at pre-therapy, Day 1and Day 3 [53], a linear model developed by pooling data from 20 Thorough QT (TQT) studies described the concentration-QTc relationship with a mean slope of 3.1 (2.8 – 3.3) ms per µg/mL moxifloxacin [54]. This is consistent with ICH E14 analyses conducted in TQT studies where maximum effect is typically within the time interval of maximum concentration [55]. In contrast, Malik *et al. *observed that peak moxifloxacin concentrations were poorly correlated with QTcF prolongation [19]. In this case, it is important to note that variability in observed moxifloxacin concentrations at *C*_max _is less than the variability in QT prolongation and that a linear-QTc prolongation relationship was identified for moxifloxacin. Tsikouris *et al*, also observed a very small change between the QTc at baseline and at 2 hours post-dose, the pharmacokinetic peak concentration of moxifloxacin [56]; however, it was not manifested in clinical symptoms.

A black-box warning statement was introduced on the moxifloxacin label warning that the duration of intravenous infusion should not be shorter than 60 minutes and the daily IV and PO recommended dose should not exceed 400 mg.

Most reports indicating that moxifloxacin may be related to cardiac AEs such as TdP are single case reports and very often the individual had multiple risk factors for cardiac arrhythmias [57, 58]. This type of case-by-case reporting serves to highlight potential cardiac risks, but cannot be used to assess the true risk of uncommon or rare cardiac events within a large patient population. One way to do this is for all clinicians to monitor and report adverse drug events to the regulatory authorities. However, to allow the authorities to obtain a true picture of the level of risk, it is important that AE reporting is standardized to include all relevant patient information, including all likely independent risk factors [52].

The benefit of using pooled randomized active-controlled clinical trial data for this type of safety study is that risk data from large numbers of patients treated with moxifloxacin can be compared with that from appropriate control subjects and comparator antimicrobials. This allows the incidence of relatively rare effects to be determined and the repeatability of relatively small changes to be analyzed. Furthermore, the data benefit from being from randomized studies and therefore patients in the two treatment groups should be balanced with respect to known, and also unknown, factors that could be associated with the outcome variables, thus making comparisons between treatment groups as fair as possible [59]. However, there are limitations to using pooled clinical trial data. Firstly, the trials included highly selected patient populations. For example, many of the studies included in this analysis excluded patients with bradycardia, electrolyte disturbances or known elevated QTc at screening or those taking concomitant anti-arrhythmic medications. This means that the patient population may not be directly representative of a typical group of patients seen in the clinic. In addition, assessment of QTc was only carried out in some of the trials included in this analysis, so data are only available for this subset of patients. As data pooling was not part of the original intention, the thresholds for reporting QTc prolongation may have been slightly different between protocols and individual investigators. A further limitation is that although this pooled analysis includes a very large patient population, it may not be large enough to identify very rare events. To uncover very rare events will require ongoing assessment of data from spontaneous reports and observational studies. Finally, these data are subject to variation because they are based on single ECG observations in individual patients not on ECG profiles (ECG may not have been measured exactly at *C*_max_).

Despite these potential limitations, this study is the first analysis using a Phase II to IV clinical trial database to focus on the cardiac safety of moxifloxacin. The findings from our study are similar to those of a large post-marketing surveillance study with PO moxifloxacin in patients with ABS, AECB, and mild-to-moderate CAP [35]. This included 27,756 patients in a “real world” setting and showed that moxifloxacin was safe and well tolerated, with no evidence of an excess of treatment-associated cardiac events. One more recent post-marketing surveillance report on specific fluoroquinolones (moxifloxacin, levofloxacin and ciprofloxacin) reported a very low incidence of TdP in relation to the overall number of spontaneous adverse event reports (990,000) for these fluoroquinolones in the USA (total number of TdP with all FQs: n=127 [0.013%]; with levofloxacin= 55 [<0.01%], with moxifloxacin= 37 [<0.01%], and with ciprofloxacin=35 [<0.01%]) [15].

In conclusion, this analysis based on a large number of patients from randomized active-controlled Bayer-sponsored Phase II to IV moxifloxacin clinical trials confirms the favorable cardiac safety profile of moxifloxacin versus a range of currently available antimicrobial agents. The established small QTc interval prolongation that occurs with moxifloxacin did not translate into a higher risk of developing clinical cardiac AEs. Therefore, although moxifloxacin should not be used in patients with known pro-arrhythmic conditions [33, 34] the overall cardiac safety of moxifloxacin, orally or intravenously, appears to be comparable to that of other commonly used antibiotics. The cardiac safety profile of moxifloxacin provided in this manuscript is in accordance with safety profiles presented by other groups independently from Bayer.

## Figures and Tables

**Fig. (1):**
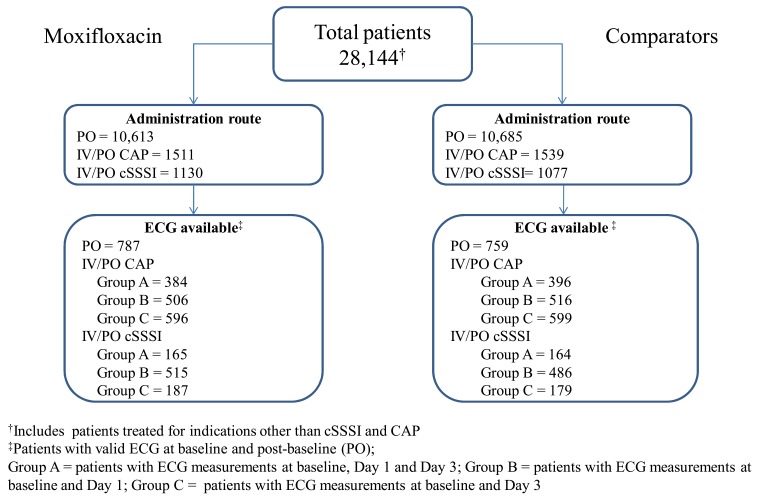
Patient Disposition Showing Numbers with Baseline and Post-Baseline ECG Data.

**Table 1.** Control Regimens Used in Phase II to IV Randomized Active-Controlled Studies with Moxifloxacin Included in this Pooled Analysis

Indication	Control Regimens
**PO studies**	Amoxicillin 500 mg t.i.d.
	Amoxicillin 1000 mg t.i.d.
	Amoxicillin 1000 mg t.i.d. + clarithromycin 500 mg b.i.d.
	Amoxicillin 500 mg/clavulanate 125 mg t.i.d.
	Amoxicillin 875 mg/clavulanate 125 mg b.i.d.
	Amoxicillin 1000 mg/clavulanate 125 mg b.i.d. + roxithromycin 150 mg b.i.d.
	Azithromycin 250 mg q.d.
	Azithromycin 500 mg (first day)/250 mg q.d. (following days)
	Cefixime 400 mg q.d.
	Ceftriaxone (IM) 1000 mg q.d.
	Cefuroxime-axetil 250 mg b.i.d.
	Cefuroxime-axetil 500 mg b.i.d.
	Cephalexin 500 mg t.i.d. ± metronidazole 400 mg t.i.d.
	Clarithromycin 250 mg b.i.d.
	Clarithromycin 500 mg b.i.d.
	Doxycycline 100 mg b.i.d. + metronidazole 400 mg t.i.d. + ciprofloxacin 500 mg (single dose)
	Levofloxacin 100 mg t.i.d.
	Levofloxacin 200 mg b.i.d.
	Levofloxacin 500 mg q.d.
	Ofloxacin 200 mg b.i.d.
	Ofloxacin 400 mg b.i.d. + metronidazole 500 mg b.i.d.
	Trimethoprim 160 mg/sulfamethoxazole 800 mg b.i.d.
	Trovafloxacin 200 mg q.d.
**IV/PO studies in CAP**	IV alatrofloxacin 200 mg q.d. then PO trovafloxacin 200 mg q.d. (initial phase)/IV levofloxacin 500 mg q.d. then PO levofloxacin 500 mg (continuation phase)
	IV amoxicillin 1000 mg/clavulanate 200 mg t.i.d. ± clarithromycin 500 mg b.i.d. then PO amoxicillin 500 mg/clavulanate 125 mg t.i.d. ± clarithromycin 500 mg b.i.d.
	IV ceftriaxone 2000 mg q.d. ± erythromycin 1000 mg t.i.d. or q.i.d.
	IV ceftriaxone 2000 mg q.d. + levofloxacin 500 mg b.i.d. then PO levofloxacin 500 mg b.i.d.
	IV ceftriaxone 2000 mg q.d. ± azithromycin 500 mg (first day)/250 mg q.d. (following days) ± metronidazole 500 mg q.i.d., then PO cefuroxime axetil 500 mg b.i.d. ± azithromycin 250 mg q.d. ± metronidazole 500 mg q.i.d.
	IV levofloxacin 500 mg q.d. then PO levofloxacin 500 mg q.d.
**IV/PO studies in cSSSI**	IV amoxicillin 1000 mg/clavulanate 200 mg t.i.d. then PO amoxicillin 500 mg/clavulanate 125 mg t.i.d.
	IV piperacillin 3000 mg/tazobactam 375 mg q.i.d. then PO amoxicillin 800 mg/clavulanate 114 mg b.i.d.
	IV piperacillin 4000 mg/tazobactam 500 mg t.i.d. then PO amoxicillin 875 mg/clavulanate 125 mg b.i.d.
**IV/PO studies in cIAI**	IV ceftriaxone 2000 mg q.d. + IV metronidazole 500 mg t.i.d. then PO amoxicillin 500 mg/clavulanate 125 mg t.i.d.
	IV piperacillin 3000 mg/tazobactam 375 mg q.i.d. then PO amoxicillin 800 mg/clavulanate 114 mg b.i.d.
**IV/PO studies in other indications^a^**	IV ampicillin 2000 mg/sulbactam 1000 mg t.i.d. then PO sultamicillin 750 mg b.i.d.
	IV ceftriaxone 2000 mg then PO cefuroxime axetil 500 mg b.i.d.

PO = oral administration; IV = intravenous administration; IV/PO = sequential IV/oral administration; IM = intramuscular; q.d. = once daily; b.i.d. = twice daily; t.i.d. = three times daily; q.i.d. = four times daily; CAP = community-acquired pneumonia; cIAI = complicated intra-abdominal infections; cSSSI = complicated skin and skin structure infections.

a Aspiration pneumonia or lung abscess, hospital-acquired pneumonia.

**Table 2.** Baseline Patient Characteristics Across All Phase II to IV Randomized Active-Controlled Studies with PO or IV/PO Moxifloxacin

	PO (N = 21,298)		IV/PO (N = 6846)	
	Moxifloxacin (N = 10,613)	Comparators^a^ (N = 10,685)	Moxifloxacin (N = 3431)	Comparators^a^ (N = 3415)
Age, mean ± SD (years)	48.2 ± 18.0	48.0 ± 17.9	56.8 ± 19.1	56.1 ± 19.2
Age ≥ 65 years, n (%)	2451 (23.1)	2403 (22.5)	1373 (40.0)	1334 (39.1)
Female sex, n (%)	5773 (54.4)	5817 (54.4)	1349 (39.3)	1323 (38.7)
Body mass index, mean ± SD (kg/m^2^)	26.0 ± 5.9	25.9 ± 5.8	26.9 ± 6.6	26.7 ± 6.4
Heart rate, mean ± SD (bpm)^b^	82.8 ± 13.6	83.0 ± 13.7	93.7 ± 18.2	93.7 ± 18.1
Cardiac disease, n (%)	1475 (13.9)	1402 (13.1)	1167 (34.0)	1136 (33.3)
Comedication known to cause QT prolongation, n (%)^c^	430 (4.1)	426 (4.0)	343 (10.0)	298 (8.7)
** Indication, n (%)**				
Acute bacterial sinusitis	2331 (22.0)	2641 (24.7)	n/a	n/a
Acute exacerbation of chronic bronchitis	4029 (38.0)	3820 (35.8)	n/a	n/a
Community-acquired pneumonia	1790 (16.9)	1822 (17.1)	1511 (44.0)	1539 (45.1)
Complicated skin and skin structure infection	n/a	n/a	1130 (32.9)	1077 (31.5)
Complicated intra-abdominal infection	n/a	n/a	618 (18.0)	622 (18.2)
Uncomplicated pelvic inflammatory disease	946 (8.9)	919 (8.6)	n/a	n/a
Other	1517^d^ (14.3)	1483^d^ (13.9)	172^e^ (5.0)	177^e^ (5.2)

PO = oral administration; IV/PO = sequential intravenous/oral administration; SD = standard deviation; n/a = not applicable.

a For a list of comparators see Table **1**.

b PO: n = 7499 moxifloxacin, n = 7170 comparators, IV/PO: n = 3401 moxifloxacin, n = 3398 comparators.

c Comedications known to cause QTc prolongation were selected according to [30] and [31].

d Uncomplicated skin and skin structure infection, complicated urinary tract infection, streptococcal pharyngitis.

e Aspiration pneumonia or lung abscess, hospital-acquired pneumonia.

**Table 3.** Treatment-Emergent Cardiac AEs: Pooled Data from Phase II to IV Randomized Active-Controlled Studies with PO or IV/PO Moxifloxacin

AE	Moxifloxacin, n^a^ (%)			Comparators^c^, n (%)		
	All	Women	≥ 65 years	All	Women	≥ 65 years
**PO studies**	**(N^b^ = 10,613)**	**(N = 5773)**	**(N = 2451)**	**(N = 10,685)**	**(N = 5817)**	**(N = 2403)**
Cardiac AE	698 (6.6)	409 (7.1)	210 (8.6)	619 (5.8)	356 (6.1)	176 (7.3)
Drug-related cardiac AE	342 (3.2)	248 (4.3)	76 (3.1)	256 (2.4)	185 (3.2)	36 (1.5)
Serious cardiac AE	75 (0.7)	21 (0.4)	42 (1.7)	72 (0.7)	27 (0.5)	35 (1.5)
Serious drug-related cardiac AE	7 (< 0.1)	3 (< 0.1)	3 (0.1)	6 (< 0.1)	3 (< 0.1)	3 (< 0.1)
Cardiac AE with fatal outcome	12 (0.1)	3 (0.1)	9 (0.4)	21 (0.2)	4 (0.1)	12 (0.5)
Drug-related cardiac AE with fatal outcome	0 (0)	0 (0)	0 (0)	1 (< 0.1)	0 (0)	0 (0)
**IV/PO studies**	**(N = 3431)**	**(N = 1349)**	**(N = 1373)**	**(N = 3415)**	**(N = 1323)**	**(N = 1334)**
Cardiac AE	377 (11.0)	178 (13.2)	213 (15.5)	410 (12.0)	181 (13.7)	244 (18.3)
Drug-related cardiac AE	49 (1.4)	24 (1.8)	22 (1.6)	50 (1.5)	23 (1.7)	26 (1.9)
Serious cardiac AE	117 (3.4)	50 (3.7)	83 (6.0)	118 (3.5)	49 (3.7)	83 (6.2)
Serious drug-related cardiac AE	6 (0.2)	3 (0.2)	5 (0.4)	11 (0.3)	6 (0.5)	6 (0.4)
Cardiac AE with fatal outcome	43 (1.3)	19 (1.4)	32 (2.3)	44 (1.3)	15 (1.1)	37 (2.8)
Drug-related cardiac AE with fatal outcome	1 (< 0.1)	0 (0)	0 (0)	3 (< 0.1)	1 (0.1)	3 (0.2)

AE = adverse event; PO = oral administration, IV/PO = sequential intravenous/oral administration.

a n = number of patients with event.

b N = total number of patients in group.

c For a list of comparators see Table **1**.

**Table 4.** Treatment-Emergent Events Considered Potential Surrogates for TdP/QTc Prolongation: Pooled Data from Phase II to IV Randomized Active-Controlled Studies with Moxifloxacin

System Organ Class • MedDRA^a^ Preferred Term	PO Studies, n^b^ (%)		IV/PO Studies, n (%)	

	Moxifloxacin (N^c^ = 10613)	Comparators^d^ (N = 10685)	Moxifloxacin (N = 3431)	Comparators^d^ (N = 3415)

Any event	25 (0.2)	23 (0.2)	37 (1.1)	36 (1.1)

Cardiac disorders				
• Cardiac arrest	1 (< 0.1)	1 (< 0.1)	9 (0.3)	11 (0.3)
• Cardiac flutter	2 (< 0.1)	1 (< 0.1)	0 (0)	0 (0)
• Cardiorespiratory arrest	2 (< 0.1)	1 (< 0.1)	4 (0.1)	5 (0.1)
• *Torsade de pointes*	0 (0)	0 (0)	0 (0)	1 (< 0.1)
• Ventricular arrhythmia	2 (< 0.1)	0 (0)	0 (0)	3 (< 0.1)
• Ventricular fibrillation	1 (< 0.1)	0 (0)	0 (0)	1 (< 0.1)
• Ventricular tachycardia	0 (0)	2 (< 0.1)	11 (0.3)	8 (0.2)

General disorders and administration				
• Death	2 (< 0.1)	2 (< 0.1)	2 (< 0.1)	0 (–)
• Sudden death	2 (< 0.1)	1 (< 0.1)	2 (< 0.1)	2 (< 0.1)
• Sudden cardiac death	0 (0)	0 (0)	1 (< 0.1)	0 (0.0)

Nervous system disorders				
• Loss of consciousness	4 (< 0.1)	5 (< 0.1)	0 (0.0)	1 (< 0.1)
• Syncope	9 (< 0.1)	10 (< 0.1)	6 (0.2)	5 (0.1)

Surgical and medicinal procedures				
• Resuscitation	0 (0)	0 (0)	2 (< 0.1)	0 (0)

PO = oral administration; IV/PO = sequential intravenous/oral administration.

a Standardized Medical Dictionary for Regulatory Activities (MedDRA) Query.

b n = number of patients with event.

c N = total number of patients in group.

d For a list of comparators see Table **1**.

**Table 5.** Patients with Serious Community-Acquired Pneumonia Requiring Intensive Care Support or Mechanical Ventilation: Treatment-Emergent Cardiac Events Considered Potential Surrogates for TdP/QTc Prolongation

System Organ Class • MedDRA^a^ preferred term	Moxifloxacin N^b^ = 149 n^c^ (%)	Comparators^d^N = 167 n (%)

Any event	7 (4.7)	8 (4.8)

Cardiac disorders		
• Cardiac arrest	3 (2.0)	3 (1.8)
• Cardiorespiratory arrest	0 (0)	1 (< 0.1)
• Ventricular tachycardia	3 (2.0)	4 (2.4)

Surgical and medicinal procedures		
• Resuscitation	1 (0.7)	0 (0)

a Standardized Medical Dictionary for Regulatory Activities (MedDRA) Query.

b N = total number of patients with serious CAP requiring intensive care support or mechanical ventilation.

c n = number of patients with event.

d dFor a list of comparators see Table **1**.

**Table 6.** Women and Patients ≥ 65 Years: Treatment-Emergent Events Considered Potential Surrogates for TdP/QTc Prolongation

	Women				Patients ≥ 65 years			

System Organ Class • MedDRA^a^ preferred term	PO Studies, n^b^ (%)		IV/PO Studies, n (%)		PO Studies, n^b^ (%)		IV/PO studies, n (%)	

	Moxifloxacin (N^c^ = 5773)	Comparators^d^ (N = 5817)	Moxifloxacin (N = 1349)	Comparators^d^ (N = 1323)	Moxifloxacin (N = 2451)	Comparators (N = 2403)	Moxifloxacin (N = 1373)	Comparators (N = 1334)

Any event	10 (0.2)	11 (0.2)	11 (0.8)	11(0.8)	9 (0.4)	7 (0.3)	24 (1.7)	22 (1.6)

Cardiac disorders								
• Cardiac arrest	0 (0)	1 (< 0.1)	4 (0.3)	4 (0.3)	1 (< 0.1)	0 (0)	4 (0.3)	6 ( 0.4)
• Cardiac flutter	1 (< 0.1)	0 (0)	0 (0)	0 (0)	1 (< 0.1)	1 (< 0.1)	0 (0)	0 (0)
• Cardiorespiratory arrest	1 (< 0.1)	1 (< 0.1)	1 (< 0.1)	2 (< 0.1)	2 (< 0.1)	1 (< 0.1)	2 (0.1)	5 (0.4)
• *Torsade de pointes*	0 (0)	0 (0)	0 (0)	1 (< 0.1)	0 (0)	0 (0)	0 (0)	1 (< 0.1)
• Ventricular arrhythmia	2 (< 0.1)	0 (0)	0 (0)	0 (0)	1 (< 0.1)	0 (0)	0 (0)	3 (0.2)
• Ventricular fibrillation	0 (0)	0 (0)	0 (0)	0 (0)	1 (< 0.1)	0 (0)	0 (0)	0 (0)
• Ventricular tachycardia	0 (0)	1 (< 0.1)	4 (0.3)	1 (< 0.1)	0 (0)	1 (< 0.1)	9 (0.7)	3 (0.2)
• Sudden cardiac death	0 (0)	0 (0)	0 (0)	0 (0)	0 (0)	0 (0)	1 (< 0.1)	0 (0)

General disorders and administration								
• Death	0 (0)	0 (0)	1 (< 0.1)	0 (0)	0 (0)	0 (0)	2 (0.1)	0 (0)
• Sudden death	0 (0)	0 (0)	0 (0)	2 (< 0.1)	1 (< 0.1)	1 (< 0.1)	2 (0.1)	2 (0.1)

Nervous system disorders								
• Loss of consciousness	1 (< 0.1)	1 (< 0.1)	0 (0)	1 (< 0.1)	0 (0)	1 (< 0.1)	0 (0)	0 (0)
• Syncope	5 (< 0.1)	7 (0.1)	1 (< 0.1)	0 (0)	2 (< 0.1)	2 (< 0.1)	4 (0.3)	3 (0.2)

PO = oral administration; IV/PO = sequential intravenous/oral administration.

a Standardized Medical Dictionary for Regulatory Activities (MedDRA) Query.

b n = number of patients with event.

c N = total number of patients in group.

d For a list of comparators see Table **1**.

**Table 7.** Summary of QTcB and QTcF Changes in Randomized Active-Controlled Phase II to IV Studies where ECG was Recorded at Baseline and Post-Baseline

	Antimicrobial	Patient Group^a^	Number of Patients with Valid ECG Measurement	Time at which QTc Measured	Heart Rate (Beats Per Min) (Mean ± SD)	QTcB (ms) Baseline (Mean ± SD)	QTcB (ms) Change (Mean ± SD)	QTcF (ms) Baseline (Mean ± SD)	QTcF (ms) Change (Mean ± SD)
**PO studies**	Moxifloxacin	n/a	787	Baseline	77.2 ± 15.4	421.4 ± 26.9		405.5 ± 27.4	
				Post-baseline	75.4 ± 13.6		6.4 ± 25.5		7.5 ± 24.8
	Comparators^b^	n/a	759	Baseline	77.0 ± 16.6	420.8 ± 27.2		405.2 ± 27.1	
				Post-baseline	74.8 ± 13.7		0.6 ± 23.1		2.0 ± 21.8
**IV/PO studies in CAP**	Moxifloxacin	A	384	Baseline	92.5 ± 17.6	418.8 ± 30.8		390.9 ± 29.8	
				Day 1	89.6 ± 16.6		10.1 ± 21.9		11.4 ± 21.4
				Day 3	81.4 ± 15.0		6.6 ± 24.7		14.5 ± 23.6
		B	506	Baseline	93.0 ± 17.5	419.9 ± 30.7		391.7 ± 29.9	
				Day 1	90.1 ± 16.7		9.8 ± 21.9		11.1 ± 21.0
		C	596	Baseline	90.5 ± 17.2	421.0 ± 32.5		394.4 ± 31.4	
				Day 3	80.1 ± 14.7		5.7 ± 26.3		13.3 ± 25.6
	Comparators^b^	A	396	Baseline	91.8 ± 17.4	420.0 ± 27.6		392.5 ± 27.5	
				Day 1	88.9 ± 16.7		–1.3 ± 20.1		0.8 ± 18.4
				Day 3	81.0 ± 14.4		–3.9 ± 26.5		4.2 ± 25.7
		B	516	Baseline	91.9 ± 17.6	419.5 ± 28.9		392.0 ± 28.5	
				Day 1	89.1 ± 16.8		–1.1 ± 19.7		0.9 ± 18.2
		C	599	Baseline	90.9 ± 16.7	421.2 ± 28.9		394.2 ± 28.5	
				Day 3	80.8 ± 14.7		–3.5 ± 27.6		4.3 ± 26.8
**IV/PO studies in cSSSI**	Moxifloxacin	A	165	Baseline	81.8 ± 15.6	413.6 ± 24.7		394.0 ± 23.6	
				Day 1	81.7 ± 14.1		6.1 ± 20.2		5.8 ± 17.0
				Day 3	75.3 ± 12.3		5.0 ± 20.6		9.9 ± 19.5
		B	515	Baseline	80.6 ± 14.4	414.0 ± 23.6		395.2 ± 22.3	
				Day 1	80.0 ± 14.2		8.7 ± 16.8		8.9 ± 14.6
		C	187	Baseline	82.2 ± 15.9	414.9 ± 26.8		394.9 ± 25.9	
				Day 3	75.0 ± 12.4		4.4 ± 21.5		9.9 ± 20.1
	Comparators^b^	A	164	Baseline	79.6 ± 14.7	412.5 ± 26.3		394.5 ± 25.0	
				Day 1	78.8 ± 13.4		1.6 ± 17.0		2.2 ± 14.0
				Day 3	73.6 ± 11.9		–3.2 ± 20.2		1.8 ± 17.6
		B	486	Baseline	80.0 ± 14.3	413.6 ± 25.8		395.3 ± 24.0	
				Day 1	78.3 ± 13.2		1.8 ± 14.7		3.1 ± 12.3
		C	179	Baseline	80.2 ± 14.7	412.2 ± 25.9		393.8 ± 24.6	
				Day 3	73.7 ± 11.9		–3.6 ± 20.2		1.8 ± 17.3

ECG = electrocardiographic; SD = standard deviation; PO = oral administration; IV/PO = sequential intravenous/oral administration; CAP = community-acquired pneumonia, cSSSI = complicated skin and skin structure infection; n/a = not applicable.

a Group A = patients with ECG measurements at baseline, Day 1 and Day 3; Group B = patients with ECG measurements at baseline and Day 1; Group C = patients with ECG measurements at baseline and Day 3.

b For a list of comparators see Table **1**.

**Table 8.** QTcB Changes in Patient Subgroups at Higher Risk of Cardiac Events

Indication	Patient Group^a^	Time QTc Measured Post-Dose	Female Patients				Patients ≥ 65 years old			
			Moxifloxacin		Comparators^b^		Moxifloxacin		Comparators^b^	
			N	QTcB Change (ms) Mean ± SD	N	QTcB Change (ms) Mean ± SD	N	QTcB Change (ms) Mean ± SD	N	QTcB Change (ms) Mean ± SD
**PO studies**	n/a	Post-baseline	434	7.2 ± 25.3	416	0.8 ± 24.1	136	1.8 ± 28.3	115	0.8 ± 25.5
**IV/PO studies in CAP**	A	Day 1	158	12.7 ± 22.3	181	0.7 ± 20.3	179	12.4 ± 21.9	170	–2.6 ± 22.0
	A	Day 3	158	7.8 ± 24.3	181	–2.1 ± 26.5	179	7.6 ± 24.4	170	–4.0 ± 27.4
	B	Day 1	205	12.4 ± 21.8	226	0.4 ± 20.2	248	10.8 ± 22.4	227	–2.5 ±21.4
	C	Day 3	239	6.7 ± 27.2	260	–3.4 ± 27.2	286	6.7 ± 26.1	281	–3.2 ± 29.0
**IV/PO studies in cSSSI**	A	Day 1	54	7.4 ± 19.8	58	–1.3 ± 17.4	33	8.2 ± 24.4	45	1.1 ± 22.4
	A	Day 3	54	7.3 ± 18.7	58	–8.7 ± 22.0	33	6.0 ± 22.0	45	1.5 ± 21.8
	B	Day 1	184	10.4 ± 16.5	164	1.5 ± 14.8	111	6.8 ± 18.0	118	2.8 ± 17.4
	C	Day 3	62	6.2 ± 21.0	60	–8.4 ± 21.8	37	6.9 ± 22.3	49	1.6 ± 21.1

SD = standard deviation; PO = oral administration; IV/PO = sequential intravenous/oral administration; CAP = community-acquired pneumonia; cSSSI = complicated skin and skin structure infections; n/a = not applicable.

a Group A = patients with ECG measurements at baseline, Day 1 and Day 3; Group B = patients with ECG measurements at baseline and Day 1; Group C = patients with ECG measurements at baseline and Day 3.

b For a list of comparators see Table **1**.
